# Ti_3_C_2_T_*x*_ MXene Thin Films and Intercalated Species Characterized by IR-to-UV Broadband Ellipsometry

**DOI:** 10.1021/acs.jpcc.4c06906

**Published:** 2024-12-27

**Authors:** Andreas Furchner, Tetiana Parker, Vincent Mauchamp, Simon Hurand, Julian Plaickner, Jörg Rappich, Aline Alencar Emerenciano, Karsten Hinrichs, Yury Gogotsi, Tristan Petit

**Affiliations:** † Nanoscale Solid−Liquid Interfaces, Helmholtz-Zentrum Berlin für Materialien und Energie GmbH, Schwarzschildstraße 8, 12489 Berlin, Germany; ‡ A.J. Drexel Nanomaterials Institute and Department of Material Science and Engineering, Drexel University, 3141 Chestnut Street, Philadelphia, Pennsylvania 19104, United States; § Université de Poitiers, CNRS, ISAE-ENSMA, PPRIME, Poitiers F-86073, France; ∥ Technische Universität Berlin, Hardenbergstraße 36, 10623 Berlin, Germany; ⊥ Helmholtz Young Investigator Group, Electrocatalysis: Synthesis to Devices, Helmholtz-Zentrum Berlin für Materialien und Energie GmbH, Schwarzschildstraße 8, 12489 Berlin, Germany

## Abstract

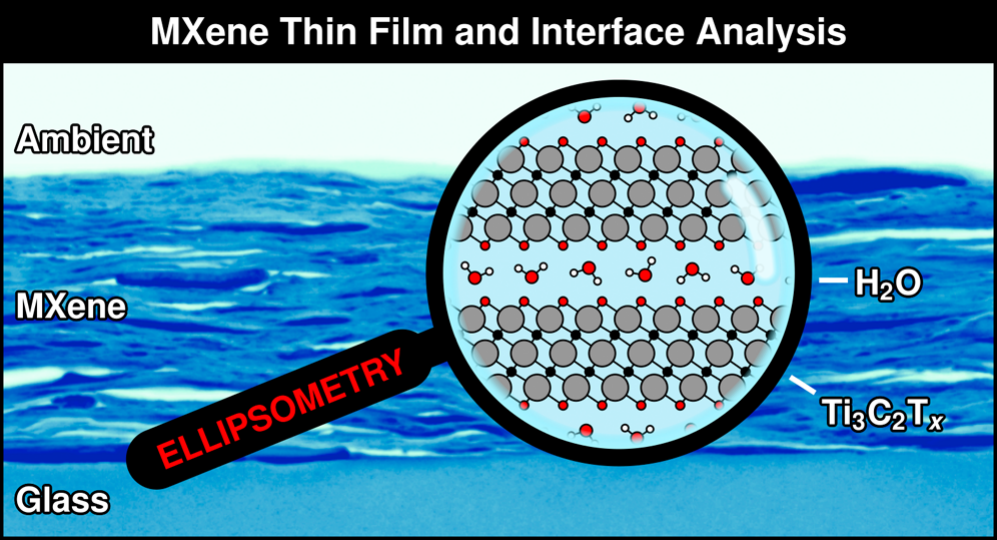

MXenes are two-dimensional (2D) materials with versatile applications in optoelectronics, batteries, and catalysis. To unlock their full potential, it is crucial to characterize MXene interfaces and intercalated species in more detail than is currently possible with conventional optical spectroscopies. Here, we combine ultra-broadband ellipsometry and transmission spectroscopy from the mid-infrared (IR) to the deep-ultraviolet (UV) to probe quantitatively the composition, structure, transport, and optical properties of spray-coated Ti_3_C_2_T_*x*_ MXene thin films with varying material properties. We find film thickness heterogeneity and surface roughness in the low-nanometer range as well as depth-dependent conductivity properties, which we quantify with a graded Drude model. The optically determined sheet resistance is confirmed by four-point probe measurements. Furthermore, we employ density-functional-theory calculations to assign the observed absorption bands in the MXene dielectric function to various interband transitions from mixed MXene surface terminations. The prominent 1.48 eV (833 nm) spectral feature is found to be related to oxygen termination. Additional plasmonic effects are also suggested. Finally, we leverage the chemical sensitivity of state-of-the-art IR ellipsometry to separate the fingerprints of intercalated species within the MXene from the dominant Drude contributions, presenting for the first time a set of infrared optical constants of intercalated water. This work lays the foundation for optical metrology for interface engineering of MXene and other 2D materials.

## Introduction

Two-dimensional transition-metal carbides and nitrides, so-called MXenes, have attracted great attention over the last decade. Their metallic conductivity and facile processability have led to new applications such as conductive transparent films, electromagnetic interference shielding, sensors, and micro-supercapacitors.^1−3^ MXenes can also exhibit electrochromic properties,^4^ which may find applications for smart windows. These properties are also highly relevant for catalysis and battery research, such as monitoring redox processes in MXene-based energy-storage devices.^5^

It is well-established that the physical and optical properties of MXene thin films are highly dependent on the synthesis method, surface chemistry, and film preparation. In the case of the most studied MXene, Ti_3_C_2_T_*x*_, it was found that intercalated species and the film deposition technique have a strong impact on the MXene electrochemical properties,^6^ while the long-term film stability depends on the defect density but also the amount of confined water within the system.^7^ The development of nondestructive techniques that enable the quantitative characterization of the electronic and optical properties of MXene thin films and intercalated species is urgently needed.

Numerous open questions remain regarding Ti_3_C_2_T_*x*_ thin films. For example, how do the optical constants depend on film thickness?^8,9^ How do film roughness and non-uniformity influence the optical response? What is the nature of the characteristic ≈800 nm (1.5 eV) spectral feature (plasmonic/interband)?^10−14^ How do intercalated species like water and ions impact the optical response?

Broadband ellipsometry^15,16^ is the spectroscopic method of choice to provide well-founded answers to such optics-related questions. Interpreting and quantifying the optical response of MXene thin films requires an adequate model of the sample’s dielectric as well as structural properties. The dielectric properties include intraband transitions responsible for the film’s Drude conductivity seen in the mid- and near-infrared (MIR, NIR) and interband transitions that give rise to a plethora of absorption features in the ultraviolet–visible (UV–Vis) range. The structural properties comprise film thickness, surface roughness, and heterogeneity (lateral film inhomogeneity). Additionally, adsorbed water from the ambient atmosphere, intercalated species (ions, molecules), and solvent residues from the film synthesis may impact the optical response.^17^

A proper model includes many structural, compositional, and electronic details that enable the determination of numerous film characteristics beyond mere effective optical film properties. Such a model can also be applied to (semi)transparent films thinner than ≈100 nm, even if they exhibit pronounced roughness and/or inhomogeneity, which commonly occur in MXenes films prepared by spin-coating, drop-casting, spray-coating, or vacuum filtration.^18^ Using simplistic models, on the other hand, can lead to a (substantial) mismatch between ellipsometric and complementary data from, e.g., transmission spectroscopy, causing errors in the extracted optical and structural MXene properties.

Original work on UV–Vis–NIR ellipsometry of spin-coated Ti_3_C_2_T_*x*_ films indicates a pronounced thickness dependence of the MXene’s optical constants,^8^ whereas other authors claim the opposite.^9^ In these works, only the tail end of the Drude response in the near-IR could be studied due to a limited spectral range. Extending the spectral range to the mid-IR would facilitate a more detailed analysis of the film’s charge-carrier properties. Furthermore, it remains unclear how intercalated water impacts the optical interpretation.

Here, we perform broadband ellipsometry (from the mid-IR to the deep-UV) of spray-coated Ti_3_C_2_T_*x*_ thin films, with complementary transmission absorption measurements crucial for decoupling optical and conductivity film properties from the structural film characteristics. Employing a graded Drude model with respect to the conductivity parameters enables us to quantify the depth-dependent dielectric function of the films. We compare the results with *ab initio* density functional theory (DFT) calculations under different MXene terminations to assign the observed electronic transitions in the UV–Vis. Furthermore, we leverage the amplitude, phase, and depolarization information gathered by ellipsometry to describe microscopic and macroscopic heterogeneity effects in the form of surface roughness and thickness non-uniformity. Lastly, we reveal the film’s chemical composition by quantifying the optical constants of the intercalated water species. This is achieved by modeling the water-associated structural heterogeneity as evidenced by the corresponding vibrational fingerprint in infrared ellipsometry spectra.

Our findings have wide implications for fundamental MXene research and applications. A resistivity gradient is highly relevant for all applications that require electrical conductivity in MXene films, such as optoelectronic devices, transparent conductive thin films, and electromagnetic interference shielding. Given the influence of intercalated water on the MXene material properties, quantifying the intercalated species is important for controlling the thin-film properties and optimizing the material for applications.

## Methods

### Film Preparation

Ti_3_C_2_T_*x*_ synthesis and thin-film preparation via spray-coating were performed according to previous protocols,^19,20^ with 1) sintering of the precursor Ti_3_AlC_2_ MAX phase under argon flow at 1400 °C, 2) MXene formation via wet chemical etching of the MAX phase using mixed acids with a HCl:H_2_O:HF ratio of 6:3:1 by volume, 3) delamination with 1 g LiCl in 20 mL deionized H_2_O to obtain Ti_3_C_2_T_*x*_ MXene flakes, and 4) spray-coating of a MXene dispersion in deionized water onto microscope-grade glass slides (Unifrog, China, pretreated with oxygen plasma).

### Ellipsometry, Transmission, and Raman Spectroscopy

Comprehensive ellipsometric and transmission measurements at room temperature were conducted in the Application Lab for Infrared Ellipsometry and in the SENTECH Thin Film Metrology Application Lab. A custom-built Mueller-matrix ellipsometer,^21^ attached to an FTIR spectrometer (Bruker Tensor 37), was employed for high-sensitivity mid-IR measurements between 14.29–1.43 μm (700–7000 cm^–1^). The ellipsometer’s photovoltaic mercury–cadmium–telluride detector (MCT, KLD-1-J1-3/11, Kolmar Technologies, USA) provides a linear response with orders of magnitude higher detectivity than standard pyroelectric detectors. Transmission (25.00–1.43 μm; 400–7000 cm^–1^) was measured in the sample compartment of the FTIR. Additional mid-IR data down to 25 μm (400 cm^–1^) were acquired with an FTIR-based ellipsometer (SENDIRA, SENTECH Instruments). NIR–Vis–UV ellipsometry and transmission measurements (SENresearch 4.0, SENTECH Instruments) were conducted between 2.50 μm and 0.20 μm. Transmission measurements were taken at normal incidence, whereas ellipsometric Ψ and Δ spectra were acquired at multiple incidence angles between 45° and 75°.

Data evaluation of ellipsometry and transmission spectra from the different instruments and spectral ranges (UV–Vis, NIR, MIR) was performed by Levenberg–Marquardt fitting, minimizing the reduced χ^2^, i.e., the sum of all error-weighted squared differences between measured and calculated data points. Before MXene analysis, broadband reference measurements were performed to thoroughly characterize the optical properties and surface roughness of the uncoated, 200 μm thick glass slides. Here, and for the subsequent MXene analysis, the treatment and incorporation of glass backside reflections and characteristics of the optical set-ups (opening angles, beam diameters, apertures, etc.) were implemented as previously described.^22^

Confocal Raman spectroscopy (LabRAM, Dilor/Horiba, ×100 microscope) was performed using the 458 nm line of a 0.6 mW Argon-ion laser.

### Electrical Measurements

Sheet resistance was measured on multiple sample spots using a four-point probe (Ossila).

### Density Functional Theory

DFT simulations were performed with the APW+lo program WIEN2k, i.e., an all-electron and full-potential approach.^23,24^ Exchange and correlation effects were treated in the Generalized Gradient Approximation (GGA) using the Perdew–Burke–Ernzerhof functional.^25^ Ti_3_C_2_T_2_ ideal systems were considered with T being O, Cl, F, or bare Ti_3_C_2_. These systems were described as periodic stacks of MXene layers, considering hexagonal unit cells (*P*6_3_/*mmc* space group) consisting of two MXene layers with the MXene’s T groups in their most stable position (fcc). Atomic positions were fully relaxed, keeping in-plane unit-cell parameters to a value of 3.058 Å (as deduced from Rietveld refinements^26^) and the out-of-plane parameter fixed to 25 Å, corresponding to an interlayer spacing of one intercalated water layer. The plane wave basis set was defined using an *RK*_max_ value of 8, except for OH terminations, where it was reduced to 4 due to the shorter O–H bonds. The muffin-tin radii associated with the different elements were obtained from an automated WIEN2k routine. Dielectric function calculations were performed with the OPTIC package^27^ in the Random Phase Approximation considering 10000 k points in the full Brillouin zone and a 0.5 eV Lorentzian broadening.

## Results and Discussion

**Figure 1:**
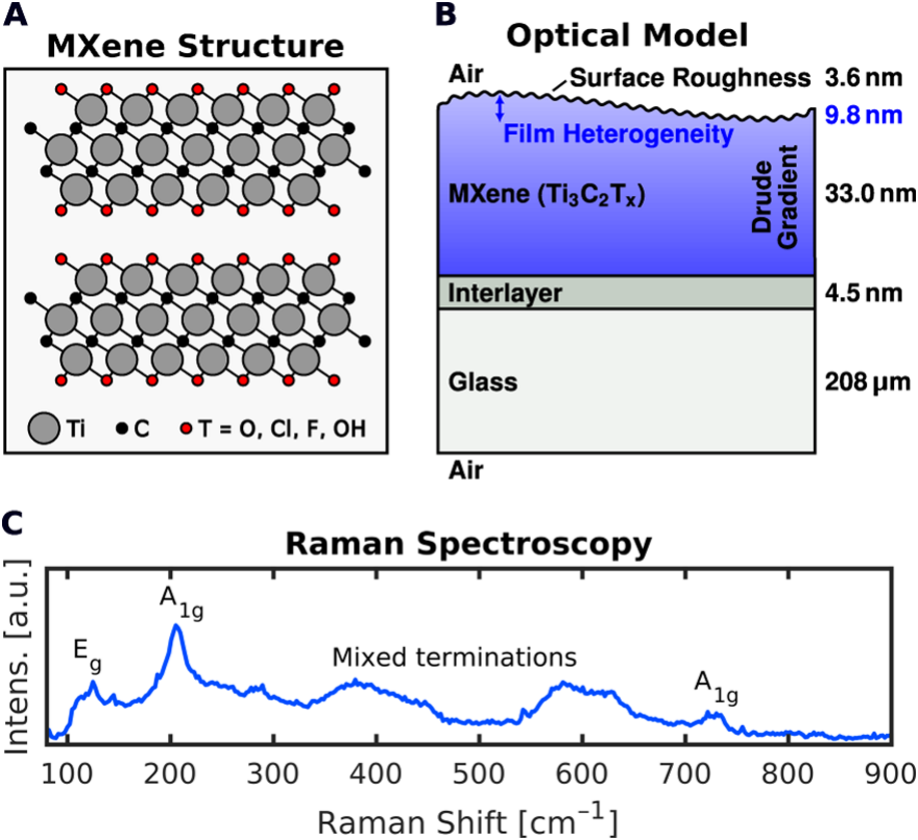
(A) Ti_3_C_2_T_*x*_ MXene structure. (B) Optical model of spray-coated MXene thin films on glass. The layer thicknesses correspond to the fitted values from the ellipsometric data analysis. (C) Raman spectrum of a 33 nm thick, spray-coated Ti_3_C_2_T_*x*_ film on glass.

The structure of Ti_3_C_2_T_*x*_ is shown in Figure 1A. As per the synthesis protocol, the MXene exhibits mixed surface functionalities (O, Cl, F, and OH).^1^Figure 1B depicts the optical layer model used for the ellipsometric analysis of spray-coated films on glass. Details of the model will be explained later.

### Raman Spectroscopy

Figure 1C shows the Raman signature of Ti_3_C_2_T_*x*_ obtained from a spray-coated MXene film on glass. We observe the expected characteristic Raman bands at 125 cm^–1^ (*E*_*g*_) and 205 cm^–1^ (*A*_1*g*_) attributed to vibrations of the Ti–C layer, at 730 cm^–1^ (*A*_1*g*_) associated with C-related vibrations, and the typical broad spectral features from 300–650 cm^–1^ recently assigned to mixed surface terminations,^28−30^ in accordance with our synthesis protocol.

### Broadband Ellipsometry

**Figure 2:**
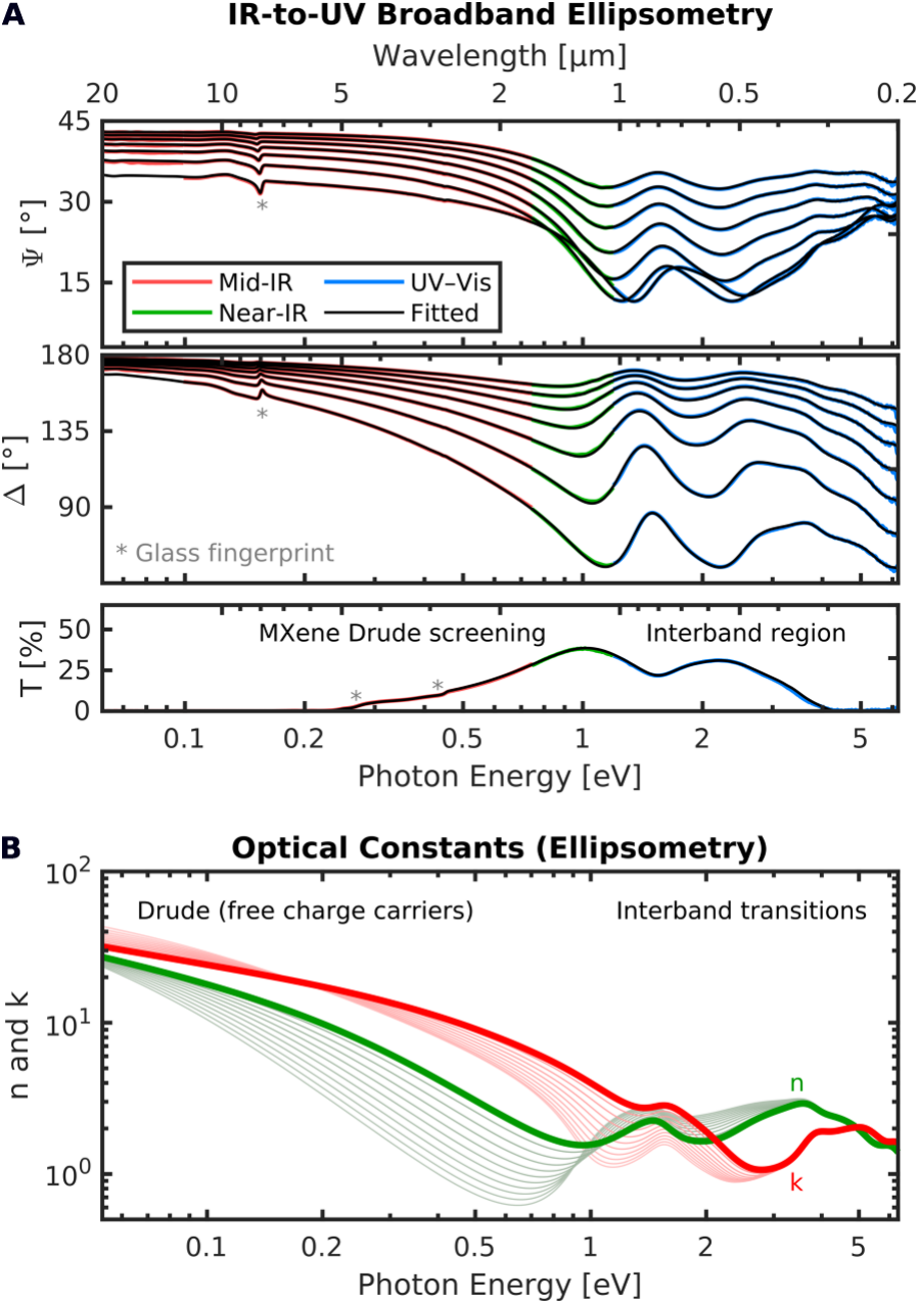
(A) Measured and fitted multiangle (45°:5°:75°, top to bottom) broadband ellipsometry (Ψ, Δ) and transmission (T) spectra of a 33 nm thick Ti_3_C_2_T_*x*_ film on glass. (B) Fitted optical constants (refractive index *n* and absorptive index *k*). Thick lines refer to values at the top of the layer, and thin lines to those toward the bottom.

Figure 2A shows measured and fitted multiangle broadband ellipsometry (amplitude Ψ, phase Δ) and transmission (T) spectra of the spray-coated Ti_3_C_2_T_*x*_ MXene thin film on glass. The optical response of the MXene film is dominated by the pronounced Drude behavior from free charge carriers in the infrared below 1.0 eV and by several absorption features related to electronic transitions in the NIR–Vis–UV. The film thickness of 33 nm was chosen such that the MXene film remains transparent throughout the IR (despite the strong Drude screening), as seen by the glass signatures around 8 μm, 5 μm, and 3 μm.^22^ Although transmission is vanishingly small below 5.5 μm (0.23 eV) due to strong absorption within the glass, these signatures provide a large optical contrast at the MXene/glass interface and, therefore, high sensitivity of the ellipsometry data toward the film properties close to the substrate. In fact, the ellipsometric measurements probe the complete film depth, allowing a sensitive fit on film thickness and depth-dependent Drude parameters.

Fit parameters of the optical model are the MXene film’s structural properties (MXene-related layer thicknesses, roughness, and thickness heterogeneity), as well as its optical and Drude parameters. The latter give rise to the energy-dependent IR-to-UV complex dielectric function ϵ(*E*) = *∑*_*j*_ϵ_*j*_(*E*) + ϵ_Drude_(*E*), described by Lorentz oscillator contributions ϵ_*j*_(*E*) = [*A_j_B_j_E_j_*]/[*E*_*j*_^2^ – *E*^2^ – *iB_j_E*] from electronic transitions (*E* = photon energy; *A*_*j*_ = amplitude, *B*_*j*_ = broadening, *E*_*j*_ = center energy) and a Drude contribution ϵ_Drude_(*E*) = −ℏ^2^/[ϵ_0_ρ(τ*E*^2^ + *i*ℏ*E*)] parameterized by the free-charge-carrier related resistivity ρ and mean scattering time τ (ϵ_0_ = vacuum permittivity, ℏ = reduced Planck constant). In agreement with atomic-force-microscopy (AFM) measurements, our optical model includes a surface-roughness layer of a few nm, described by a Bruggeman effective medium approximation (EMA) that mixes 50% MXene with 50% air. Furthermore, we accounted for the macroscopic film heterogeneity of several nm (within the measurement spot) that is inherent to the film coating process. This heterogeneity causes depolarization (accessible and quantifiable by ellipsometry^21,31^) and a broadening/smearing of otherwise sharper absorption and film-interference features in the amplitude, phase, and transmission spectra. Including roughness and heterogeneity is essential to accurately describing ellipsometry and transmission data simultaneously, and thereby to extracting meaningful and reliable material properties. Surface roughness and film thickness heterogeneity are found to be in the low-nanometer range, demonstrating the high film quality that renders spray-coating attractive for scalable manufacturing.

We also find that the observed Drude response in the MIR–NIR range cannot be reproduced by a model with constant film conductivity. To describe the optical response toward the far-infrared, a small gradient (with film depth) in the Drude parameters ρ and τ of the MXene layer has to be included. Such a gradient agrees with previously reported thickness-dependent near-IR optical constants of spin-coated Ti_3_C_2_T_*x*_ thin films,^8^ especially in the ultrathin film regime below 30 nm.

**Figure 3:**
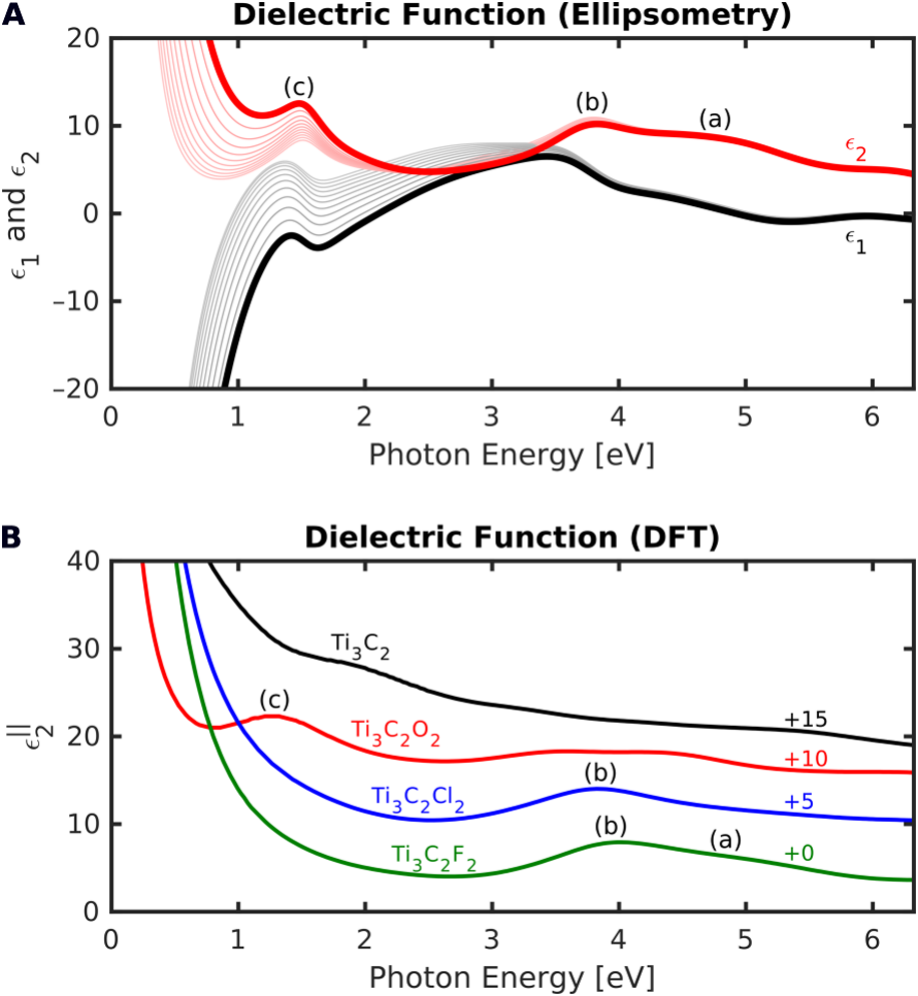
(A) Real part ϵ_1_ and imaginary part ϵ_2_ of the Ti_3_C_2_T_*x*_ dielectric function obtained from ellipsometry. The thick lines refer to values at the top of the layer. Marked transitions (a), (b), and (c) are discussed in the text. (B) *Ab initio* DFT calculations of ϵ_2_^∥^ for different surface terminations.

The resulting fitted optical constants (refractive index *n* and absorptive index *k*) and dielectric function ϵ = ϵ_1_ + *i*ϵ_2_ = (*n* + *ik*)^2^ are shown in Figures 2B and 3A, respectively. Their depth dependence, indicated with thin lines, substantiates the thickness-dependent trend in ϵ seen by Dillon et al.^8^ The Ti_3_C_2_T_*x*_ film exhibits metallic behavior, with the real part ϵ_1_ being negative up to about 1.3 eV, rendering the material system highly relevant for NIR plasmonics applications.^8,32^ Based on the optical model, the characteristic band contributions for Ti_3_C_2_T_*x*_ can be extracted. We find strong bands at 5.01 eV [247 nm] and 4.43 eV [280 nm] (a), 3.76 eV [329 nm] (b), and 1.48 eV [837 nm] (c), as well as two weaker ones around 3.01 eV [411 nm] and 1.89 eV [656 nm]. Further absorption features outside the measured spectral range are indicated by the broad absorption and dispersion tails above 5 eV.

### DFT Calculations

To better interpret the ellipsometrically determined dielectric function, we performed *ab initio* DFT calculations of ideal MXene structures. The in-plane component ϵ_2_^∥^ of the dielectric tensor was calculated for different surface terminations (none, O, Cl, F, OH) and is shown in Figure 3B for bare, O-, Cl-, and F-terminated MXene. Calculations with OH termination (not shown) are similar to those with F termination.

The double structure at 4.8 eV (a) and 3.9 eV (b) can be attributed to F and OH groups, with Cl groups also contributing to the (b) feature, in accordance with our MXene synthesis. These peaks are related to electronic transitions between C-p/Ti-d states in the valence and Ti-d states in the conduction band.^26^ F and O electronic states are only very weakly involved, but the T_*x*_ groups affect the position of the C-p and Ti-d states with respect to the Fermi energy, thus driving the photon energy position of the corresponding interband transition in the spectrum.

The prominent feature at 1.48 eV (c) is discussed in the literature as being related to a plasmon resonance^11^ or an interband transition.^12−14^ We observe it only for the oxygen-terminated MXene (Ti_3_C_2_O_2_). Ti-d states in the conduction band very close to the Fermi level are involved in this peak, rendering its intensity highly sensitive to the Ti-d band filling. Putting F or OH groups on the MXene surface, which brings one more electron to the valence band than O groups, leads to a shift of the Fermi level and a significant reduction in peak intensity. Experimentally, position and shape of the 1.48 eV band can vary depending on sample preparation method and conditions, which points to a more complex band interpretation. Therefore, we suggest a mixed interband/plasmonic origin of the 1.48 eV feature, given the sign change in ϵ_1_ close to the resonance energy, as well as the micrometer-sized dimensions of the MXene flakes within the film that could lead to intrafilm and surface roughness effects promoting a corresponding (local) surface plasmon resonance. Such a mixing could imply the possibility of a coupling between surface plasmons and interband transitions.^33^ Considering the key role played by interband transitions on surface plasmon damping when plasmons and interband transitions are coupled,^34,35^ having a mixture between the two excitations reasonably explains the peak’s high sensitivity toward the MXene surface chemistry.^13,14^ This also highlights surface-chemistry engineering as a relevant approach to deeply modify such excitations in Ti_3_C_2_T_*x*_ thin films.^36^

### Graded Drude vs Four-Point Probe Conductivity

The conductivity parameters of MXene films depend both on the quality of the MXene and on the film structure and morphology, including features such as flake alignment, film roughness, and interflake distance.^19^ Using ellipsometry to analyze the Drude parameters with film depth could be a promising route for optimizing and tailoring the film properties.

**Figure 4:**
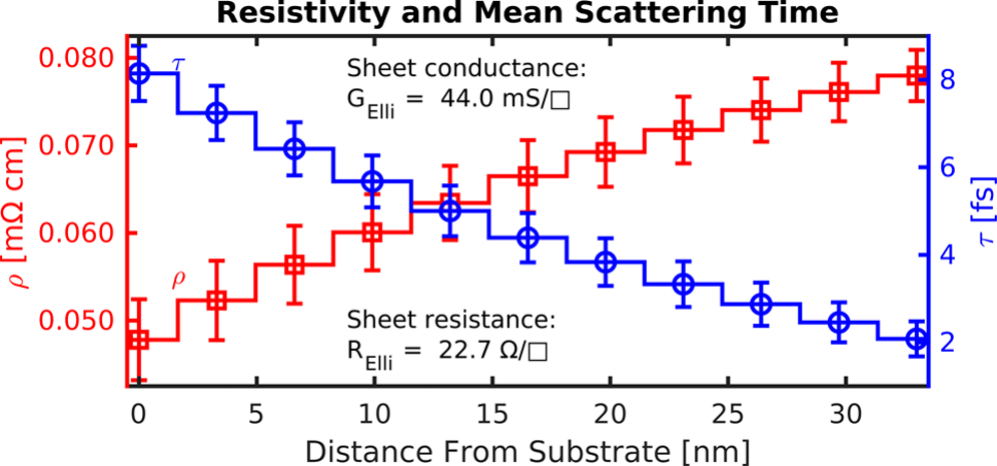
Depth-dependent Drude parameters (resistivity ρ and mean scattering time τ) from the ellipsometric fit of the Ti_3_C_2_T_*x*_ MXene film (with 11 sublayers).

The ellipsometrically determined grading profile of resistivity ρ and mean scattering time τ with film depth is shown in Figure 4. While ρ increases from about 4.7 × 10^–5^ Ω cm to 7.8 × 10^–5^ Ω cm toward the film surface, τ decreases from about 8.1 fs to 2.1 fs. This decrease in conductivity (20900 S cm^–1^ to 12800 S cm^–1^) of the spray-coated Ti_3_C_2_T_*x*_ film toward the MXene/air interface could be related to local surface oxidation or defects close to the film surface.^17,19^ The above resistivity (conductivity) values are comparable to reported literature values.^4,8,19,37,38^

To ascertain the validity of the graded Drude optical model, particularly its applicability in the infrared range, we compare the film’s sheet resistance *R* as determined optically from ellipsometry with a direct electrical measurement of the same sample spot via a four-point probe. Broadband ellipsometry allows one to calculate *R* from the fitted free-carrier parameters of the graded Drude layer, according to *R*_Elli_ = 1/(*d*_1_/ρ_1_ + *d*_2_/ρ_2_ + ... + *d*_*n*_/ρ_*n*_), where ρ_*i*_ and *d*_*i*_ are the resistivity and thickness of each sublayer, respectively, and *∑*_*i*_*d*_*i*_ = *d*_total_ is the total thickness of the MXene layer see (Figure 4). The resulting sheet resistance of *R*_Elli_ = (22.7 ± 0.8) Ω/□ agrees within a few percent with the four-point-probe measurement of *R*_4PP_ = (24.2 ± 0.5) Ω/□ corroborating the graded-Drude approach. The corresponding sheet conductances (*G* = 1/*R*) are *G*_Elli_ = (44.0 ± 1.7) mS/□ and *G*_4PP_ = (41.3 ± 0.8) mS/□.

The origin of the intrafilm resistivity grading and its relationship with the film microstructure have yet to be elucidated. Possibly, the presence of confined water close to the surface may lead to local oxidation, thereby decreasing the conductivity.^17^ Examining the Drude grading upon variations in film preparation conditions and methods (spray-coating, spin-coating, etc.) is the aim of future studies.

### Intercalated Water

**Figure 5:**
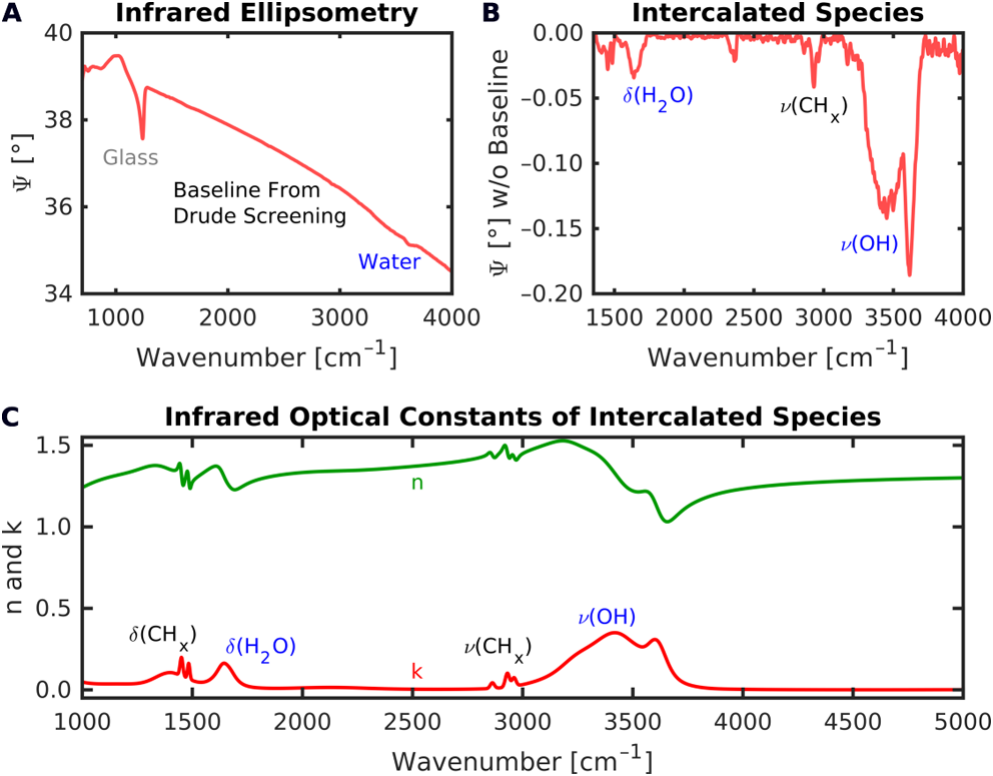
(A) Mid-IR ellipsometric Ψ spectrum measured at 65° incidence angle. (B) MXene vibrational fingerprint in the baseline-removed Ψ spectrum related to intercalated water. (C) Fitted infrared optical constants of intercalated water with CH_x_ residues. Major vibrational contributions are marked (CH_x_- and OH-related bending and stretching modes).

Overall, the ellipsometric baselines from the Drude screening in the MIR–NIR range can be adequately described by the graded Drude model. However, a closer look into the mid-IR range reveals additional vibrational structures several orders of magnitude below the dominating Drude, which require further treatment within the model. As shown in Figure 5A,B, multiple bands are found in the OH-bending (1650 cm^–1^) and -stretching (3300 cm^–1^) region that suggest the presence of water within the MXene film, with the narrow band at 3650 cm^–1^ indicating weakly bound (or “free”) OH groups. The band envelope of the OH-stretching region between 3000–3700 cm^–1^ in the baseline-removed Ψ spectrum in Figure 5B resembles that of Li-ion-intercalated Ti_3_C_2_T_*x*_ measured with ATR spectroscopy in humid air.^39^ This observation agrees with our film synthesis, which uses LiCl in H_2_O as the delamination agent. In other words, the IR ellipsometry data are consistent with the presence of water with Li ions inside the spray-coated MXene films.

However, the measured band shapes (absorption-like in Ψ and dispersion-like in Δ) cannot be reproduced with an optical model that assumes a homogeneous, isotropic mixture of MXene and water throughout the layer. This observation indicates some degree of structural anisotropy and/or heterogeneity that is overlooked when analyzing just the NIR–Vis–UV range, and that has not been reported in the literature.

A first explanation of the IR band shapes could be a thin water-rich region at the interface to the ambient atmosphere at the top of the metal-like MXene. In the corresponding structurally heterogeneous model, this hydrated MXene layer of a few nm thickness is described by a Bruggeman effective medium approximation (EMA), which mixes the conductive MXene with water^40^ and additional species in the (spherical) inclusion material, amounting to a total water content of about 10 vol.% water when projected throughout the whole MXene film. This proportion of water is consistent with an average interlayer filling of one water layer between the Ti_3_C_2_T_*x*_ sheets^41^ in the presence of Li ions.^42^ The hydrated EMA layer only marginally affects the previously discussed MXene dielectric function due to the dominating Drude free-carrier contribution. Nonetheless, the high-sensitivity mid-IR ellipsometry measurements allow us to obtain a first set of optical constants *n* and *k* of the intercalated species within the MXene that quantify the water/ion vibrational fingerprint, as demonstrated in Figure 5C. Besides the characteristic water bands, and a broad band around 1450 cm^–1^ of yet unknown origin, several modes likely related to organic residues are found in the CH_x_-bending and stretching region below 1500 cm^–1^ and 3000 cm^–1^. This finding agrees with the observation of carbon species from X-ray photoelectron spectroscopy (XPS).^43^

A second and more realistic explanation of the IR band shapes is that water and MXene are indeed homogeneously mixed (on the IR wavelength scale of μm) but the water inclusions are structurally anisotropic, as expected for confined water in Ti_3_C_2_T_*x*_.^44,45^ Such mixing gives rise to an anisotropic optical response, which can be described by an anisotropic Bruggeman effective medium approximation (AB-EMA), in accordance with the arrangement of MXene flakes within the layer that can trap water between the sheets or in free pockets/voids. An initial modeling approach based on an AB-EMA^46^ (describing ellipsoidal water inclusions) indicates that water could be included in the film in the form of oblate-shaped pockets with a height-to-width ratio of about 1:80 (or even more oblate), which is in accord with the dimensions of the MXene flakes (μm laterally, nm-sized flake thickness). Parameter correlation in the AB-EMA model is yet too high to make definitive statements here, but these first indications are promising for further controlled experiments under varying ambient humidity.

## Conclusions

We presented a comprehensive analysis of spray-coated Ti_3_C_2_T_*x*_ thin films by complementary broadband ellipsometry and transmission spectroscopy, disentangling the MXene films’ optical properties and conductivity parameters from the structural and morphological features. We found a gradient in Drude resistivity with film depth. Based on DFT, the characteristic 1.48 eV (833 nm) spectral feature in the dielectric function was assigned to oxygen termination, suggesting a dual interband/plasmonic origin. Furthermore, we quantified the infrared optical constants of intercalated water within the MXene film. A comprehensive exploration of hydration effects on the broadband dielectric function will be the subject of future studies. It is clear, however, that—besides exploring Drude gradients—the chemical sensitivity gained by including infrared measurements provides a new powerful tool for future in-depth studies of MXene compositions and water–MXene interactions.

## Data Availability

The data that support the findings of this study are available from the corresponding author upon reasonable request.
